# An Examination of the Practice of Blood-Letting in Mental Disorders

**Published:** 1854-07

**Authors:** 


ART. VII.—*An Examination of the Practice of Blood-Letting in Mental Disorders.* By PLINY EARLE, M. D., author of A Visit to thirteen Asylums for the Insane in Europe; &c., &c. NewYork: S. S. & W. Wood; 1854.

---

WE have already given, in a former number, the conclusions at which the learned writer of this work arrives with regard to the propriety of blood-letting in insanity, and can now only quote the remarks in which he relates his own experience in the disease, adding that Dr. EARLE has produced a most conclusive argument on this subject:

My first impressions in regard to the disease, were such as to induce a pretty frequent resort to topical bleeding, and occasionally to venesection. My practice became, however, essentially modified, as my knowledge of the disease increased. An accurate idea of it may be obtained from the following synopsis.

In the course of the four years from 1845 to 1848, both inclusive, the cases of most recently developed insanity, and of the first attack, received into the Bloomingdale Asylum, were as follows:

| | | | | | |
|---|---|---|---|---|---|
| 19 | cases in which the disease | had existed | from | 1 to 7 | days. |
| 11 | do | do | do | 8 to 10 | " |
| 31 | do | do | do | 11 to 14 | " |
| 14 | do | do | do | 15 to 21 | " |
| 2 | do | do | do | 22 to 28 | " |

Of these *seventy-seven* patients, eight had been bled by venesection, and *seven* by cups and leeches, before admission. Of the *eight* bled from the veins, *four* recovered and *four* died. Of the *seven* bled locally, *five* recovered and *two* were discharged in different degrees of improvement.

At the asylum, venesection was not resorted to for either of the seventy-seven patients. *Seven* of them were cupped, and *all* recovered. In one of these cases the cupping was practised as a precautionary measure against a rapidly increasing plethora, in an advanced stage of convalescence. In another, the patient had been leeched before admission. In a third, the patient was a robust, athletic, plethoric young man, a farmer, whose disease was of very sudden invasion, and violent in form. He was bled from the arm three times in the first few days. After coming to the asylum he still appeared too plethoric. I ordered cupping on the nape of the neck. When but three ounces of blood were drawn, he fainted—a confirmation of the assertion of Dr. Burrows, that prostration follows sanguine depletion even when the symptoms appear to indicate it, as well as of the alleged fact that the insane cannot tolerate this treatment as well as the sane.

The result of all the cases was as follows:—*recovered*, 52, including five discharged during convalescence, which regularly progressed to a cure; *improved*, in various degrees, 8; *unimproved*, 5; *died*, 12. Of the patients not cured, four were removed after a residence of but four, ten, seventeen, and thirty-five days, respectively, and consequently did not receive a fair trial of curative means. Several of those who died were not removed from home until much prostrated. One died in but one day after admission, one in two days, two in three days, and two in seven days. One of the remaining six died of ship fever. He was considered curable before attacked with that disease.

To illustrate the effect of experience upon my practice in regard to bleeding, even locally, the four years may be divided into two periods, and the results of treatment given.

In 1845 and '46, admitted, 32; cupped, 6; cured, 21; equal to 66⅔ per cent. In 1847 and '48, admitted, 45; cupped, 1; cured, 31; equal to 68 per cent.

Thus, of thirty-two patients received in the first period, six were cupped, while of forty-five in the last period only one was cupped, and the per centage of cures was increased.

Besides these cases, there was a considerable number, also recent, of periodical or recurrent mania. It is not essintial to give a detail of the practice in these. The general result would not be materially affected thereby. If modified at all, it would be in favor of abstinence from blood-letting in any way.

